# Triple-negative invasive breast carcinoma: the association between the sonographic appearances with clinicopathological feature

**DOI:** 10.1038/s41598-018-27222-6

**Published:** 2018-06-13

**Authors:** Jia-wei Li, Kai Zhang, Zhao-ting Shi, Xun Zhang, Juan Xie, Jun-ying Liu, Cai Chang

**Affiliations:** Department of Medical Ultrasound, Fudan University Shanghai Cancer Center, Department of Oncology, Shanghai Medical College, Fudan University, Shanghai, 200032 China

**Keywords:** Ultrasound, Molecular medicine, Breast cancer

## Abstract

In this study, we aimed to evaluate the clinical and pathological factors that associated with sonographic appearances of triple-negative (TN) invasive breast carcinoma. With the ethical approval, 560 patients who were pathologically confirmed as invasive breast carcinoma were reviewed for ultrasound, clinical, and pathological data. Logistic regression analysis was used to identify the typical sonographic features for TN invasive breast carcinomas. The effect of clinical and pathological factors on the sonographic features of TN invasive breast carcinoma was studied. There were 104 cases of TN invasive breast carcinoma. The independent sonographic features for the TN subgroup included regular shape (odds ratio, OR = 1.73, p = 0.033), no spiculated/angular margin (OR = 2.09, p = 0.01), posterior acoustic enhancement (OR = 2.09, p = 0.004), and no calcifications (OR = 2.11, p = 0.005). Higher pathological grade was significantly associated with regular tumor shape of TN breast cancer (p = 0.012). Higher Ki67 level was significantly associated with regular tumor shape (p = 0.023) and absence of angular/spiculated margin (p = 0.005). Higher human epidermal growth factor receptor 2 (HER2) score was significantly associated with the presence of calcifications (p = 0.033). We conclude that four sonographic features are associated with TN invasive breast carcinoma. Heterogeneity of sonographic features was associated with the pathological grade, Ki67 proliferation level and HER2 score of TN breast cancers.

## Introduction

The St. Gallen International Expert Consensus proposed a new classification system including Luminal A, Luminal B with HER2-, Luminal B with HER2+, HER2-enriched, and triple-negative (TN) for breast cancers based on the four immunohistochemical indices: estrogen receptor (ER), progesterone receptor (PR), human epidermal growth factor receptor 2 (HER2), and Ki67^1^. TN breast cancer, lacking of expression of ER, PR, and HER2, constitutes 10–20% of all breast cancers^2,3^. TN breast cancer is associated with younger patient age, larger tumor size, aggressive histopathological features, and lymph node involvement at diagnosis^2,4^. Treatment of TN breast cancers has been a challenge due to the heterogeneity of the disease and the absence of well-defined molecular targets. Therefore, TN breast cancer has a higher rate of recurrence and distant metastasis resulting in a poor prognosis^2–8^.

The early diagnosis of TN breast cancer is crucial in virtue of the poor prognostic outcome. However, the imaging appearances of TN breast cancer showed a great variety as a result of its biological and clinical characteristics^9–13^. Some TN breast masses especially in young patients can be misinterpreted as benign tumors due to the benign-like sonographic appearances^13^. An early and accurate recognition of this kind of breast tumor with poor prognosis will, therefore, be beneficial for preoperative planning and outcome improvement. Therefore, knowledge of the sonographic appearances of breast cancers and their possible variations determined by the tumor biology is important for the ultrasound radiologist to minimize misdiagnosis. The sonographic appearances of TN breast cancers have been reported in previous studies^9–18^. However, few of these studies addressed the heterogeneity of sonographic appearances of TN breast cancers which was the primary objective of the present study. In addition, we evaluated the association between sonographic appearances of TN breast cancer and its clinicopathological and immunohistochemical indices.

## Materials and Methods

This study was approved by the institutional review board at Fudan University Shanghai Cancer Center, and the need for informed consent from patients was waived due to the non-interventional retrospective study design. Patients who accepted breast cancer surgeries at our cancer center from June 2014 to October 2016 were reviewed for the preoperative ultrasound reports, surgical records, and postoperative pathological and immunohistochemical results. Patients who were diagnosed with invasive breast carcinoma and presented with a solitary mass on ultrasound images were eligible for the study. Patients with bilateral or multiple masses, recurrences, previous breast cancer surgeries, neoadjuvant chemotherapy or large mass lesion involving the whole breast were excluded. Patients with a poor quality of ultrasound images were also excluded. Finally, 560 eligible patients were enrolled.

### Ultrasound equipment and image assessment

Ultrasound images of the breast mass in the database were recorded from the digital imaging and communication in medicine (DICOM) output of the ultrasound equipment. We used high-end ultrasound machines including Aixplorer (Supersonic Imaging, Aix-en-Provence, France), Logic E9 (GE Healthcare, Kretz, Zipf, Austria), IU22 (Philips Medical Systems, Bothell, WA, USA), Aplio 500 (Toshiba medical system, Japan), and Mylab90 (Esaote, Genoa, Italy). All ultrasound machines were equipped with a high frequency (5–14 MHz) linear array transducer. All ultrasound images were reviewed by two experienced doctors who were experts in the areas of breast ultrasound and BI-RADS lexicon. Both doctors were blinded to the patients’ clinical characteristics and histological results. In the case of a disagreement between the two examiners, a consensus was reached after discussion.

The sonographic features of the breast carcinomas were assessed based on the BI-RADS lexicon: orientation (parallel and not parallel), shape (regular and irregular), margin (circumscribed and not circumscribed), spiculated/angular margin (yes and no), echo pattern (hypoechoic, mixed solid echo, complex cystic and solid echo), posterior acoustic pattern (shadow, enhancement and no change), and the presence of calcifications (yes and no)^19^.

### Pathology and immunohistochemistry analysis

All pathology and immunohistochemistry analysis were routinely performed with proper quality assurance by the Pathology department at our Breast Cancer Center. Breast tumors were fixed in formalin, embedded in paraffin, and stained with hematoxylin-eosin (HE) to prepare the histological specimen. The pathologist determined the size of the tumor mass from the gross sample before preparation of the histological specimen. The characteristics evaluated by HE staining included pathological type, nuclear grade, lymphatic vessel invasion, papilla invasion and lymph node metastasis. Based on pathology, invasive breast carcinomas were divided into infiltrating ductal carcinoma, infiltrating lobular carcinoma and other types of invasive breast carcinomas. Based on nuclear grades the carcinomas were divided into three groups: grade I (highly differentiated), grade II (moderately differentiated), and grade III (poorly differentiated).

The expression status of ER, PR, HER2, and Ki67 were determined by immunohistochemistry as per instructions. The cutoff for ER and PR positive expression was defined as ≥1% staining. HER2 status was graded as 0, 1+, 2+ or 3+. A score of 0 and 1+ was defined as negative, while 3+ was deemed as positive. A score of 2+ was indeterminate, which was further confirmed by fluorescent *in situ* hybridization (FISH) for possible gene amplification. TN breast cancers were defined as ER, PR, HER2 negative in accordance with the St. Gallen International Expert Consensus on the primary therapy of early breast cancer 2013^1^.

The effect of age, tumor size, pathological grade, Ki67 and HER2 on the sonographic appearances of TN invasive breast carcinoma was evaluated. Patients were divided to three groups according to age: <45 yrs, 45–60 yrs and >60 yrs; three groups according to tumor size: <2 cm, 2–5 cm, >5 cm; 2 groups according to pathological grade: low grade (I &II) and high grade (III); two groups according to Ki67 level: <40% and ≥40%; and two groups according to HER2 score: score 0 or 1 and score 2.

### Statistical analysis

Statistical analyses were performed with SPSS for Windows version 19.0 (SPSS Inc., Chicago, IL, USA). Continuous numerical data were presented as mean (standard deviation; SD). Categorical data were presented as frequency (%). Comparisons of continuous data were performed with independent samples t test. The Pearson’s chi-square test was used for comparing categorical data. Differences were considered significant at a p-value of less than 0.05.

Univariate and multivariate logistic regression analyses were used to determine the typical sonographic features for TN breast cancers. TN group was compared with the combination of non-TN breast cancers. The odds ratio (OR) and its 95% confidence interval (CI) were calculated. Meanwhile, the sensitivity, specificity, positive predictive value (PPV), and negative predictive value (NPV) were calculated for each specific sonographic feature determined by multivariate logistic regression.

### Advance(s) in Knowledge

1. Triple-negative (TN) invasive breast carcinomas present significant differences in clinical, pathological, and sonographic features compared with non-TN invasive breast carcinomas.
2. Four sonographic features are associated with TN invasive breast carcinoma.
3. Great diversity of sonographic features was found for the TN invasive breast carcinoma.
4. The pathological grade had significant effect on the tumor shape, the expression of Ki67 had significant effect on the tumor shape and presence of spiculated/angular margins, and HER2 score had significant effect on the presence of calcifications.
5. Some TN invasive breast carcinomas presented similar sonographic appearances with breast fibroadenomas.

### Implication(s) for Patient Care

1. While being recognized as an aggressive disease, an early and accurate recognition of TN breast cancer will be beneficial for preoperative planning and outcome improvement.
2. Knowledge of the sonographic appearances of breast cancers and their possible variations determined by the tumor biology is important for the ultrasound radiologist to minimize misdiagnosis.
3. Benign-like breast masses with an oval shape and without angular or spiculated margin in young patients require additional attention during ultrasound examinations, particularly by less experienced doctors.

### Summary statement

TN breast cancers had typical sonographic features. Variations of sonographic features are associated with the pathological grade, Ki67 proliferation level and HER2 score. Some TN breast cancers are easily to be misdiagnosed as benign breast masses especially for young patients.

## Results

Seventeen patients were excluded from the 560 patients due to an uncertain HER2 amplification status, following the FISH test. Finally, a database of 543 patients was created containing the demographics, ultrasound images, surgical procedures, pathological records and immunohistochemical indices. There were 104 TN and 439 non-TN breast cancers. Table 1 shows the demographics of patients and the general information of surgery and pathology in the TN and non-TN groups. Compared with non-TN breast cancer, TN breast cancer had more chance to be high pathological grade (p < 0.0005) and less chance to accompany with axillary lymph node metastasis (p = 0.021). However, no significant differences were found in terms of age (p = 0.641), tumor size (p = 0.723), surgical type (p = 0.31), pathological type (p = 0.424), lymphatic vessel invasion (p = 0.083) and papilla invasion (p = 0.221).

**Table 1:** Clinical and pathological features of the TN and non-TN invasive breast carcinoma subgroups. Data for age and size are presented as mean (SD), data for other variables are presented as frequency (%).

	TN (n = 104)	Non-TN (n = 439)	P value
Age (yrs)	52.2 (11.9)	52.8 (10.9)	0.641
Size (cm)	2.2 (0.9)	2.2 (0.9)	0.723
Side			0.853
Left	57 (54.8)	245 (55.8)	
Right	47 (45.2)	194 (44.2)	
Surgical type			0.31
MRM	42 (40.4)	118 (26.9)	
M&SLNB	29 (27.9)	113 (25.7)	
M&ALND	1 (1.0)	20 (4.6)	
BCS&SLNB	27 (26.0)	89 (20.3)	
BCS&ALND	5 (4.8)	29 (6.6)	
Pathological type			0.424
Ductal carcinoma	101 (97.1)	421 (95.9)	
Lobular carcinoma	1 (1.0)	13 (3.0)	
Others	2 (1.9)	5 (1.1)	
Grade			<0.0005
I&II (low grade)	18 (18)	239 (56.2)	
III (high grade)	82 (82)	186 (44.8)	
Axillary lymph node metastasis			0.021
yes	29 (27.9)	176 (40.1)	
no	75 (72.1)	263 (59.9)	
Lymphatic vessel invasion			0.083
yes	29 (27.9)	162 (36.9)	
no	75 (72.1)	277 (63.1)	
Papilla invasion			0.221
yes	2 (1.9)	20 (4.6)	
no	102 (98.1)	419 (95.4)	

yrs: years-old, MRM: Modified radical mastectomy, M: Mastectomy, SLNB: Sentinel Lymph Node Biopsy, ALND: Axillary Lymph Node Dissection, BCS: Breast Conservative Surgery.

In terms of sonographic features, TN breast cancer showed significant differences (p < 0.05) compared with non-TN breast cancer in the tumor shape, the presence of spiculated/angular margin, echo pattern, posterior acoustic pattern, and calcinations, as shown in Table 2. No significant differences were seen with regards to the tumor orientation (p = 0.548) and the BI-RADS score (p = 0.531).

**Table 2:** Sonographic features of the TN and non-TN invasive breast carcinoma subgroups. Data are presented as frequency (%).

Sonographic feature	TN (n = 104)	Non-TN (n = 439)	P value
Orientation			0.548
Parallel	46 (44.2)	180 (41)	
Not parallel	58 (55.8)	259 (59)	
Shape			0.017
Regular	31 (29.8)	84 (19.1)	
Irregular	73 (70.2)	355 (80.9)	
Margin			0.026
Circumscribed	14 (13.5)	30 (6.8)	
Not circumscribed	90 (86.5)	409 (93.2)	
Spiculated/angular margin			0.004
yes	18 (17.3)	138 (31.4)	
no	86 (82.7)	301 (68.6)	
Echo pattern			0.005
Hypoechoic	77 (74.0)	378 (86.1)	
Mixed solid echo	20 (19.2)	51 (11.6)	
Complex cystic and solid echo	7 (6.7)	10 (2.3)	
Posterior acoustic pattern			<0.0005
Shadow	8 (7.7)	76 (17.3)	
Enhancement	36 (34.6)	70 (15.9)	
No change	60 (57.7)	293 (66.7)	
Calcifications			0.009
yes	22 (21.2)	151 (34.4)	
no	82 (78.8)	288 (65.6)	
BI-RADS score			0.531
6	1 (1.0)	13 (3.0)	
5	28 (26.9)	133 (30.3)	
4C	52 (50.0)	193 (44)	
4B	18 (17.3)	86 (19.6)	
4A	5 (4.8)	14 (3.2)	

The univariate and multivariate logistic analyses for the prediction of characteristic sonographic appearances for TN breast cancer are shown in Tables 3 and 4. There were four independent sonographic features for the TN subgroup including regular shape (OR = 1.73, p = 0.033), no spiculated/angular margin (OR = 2.09, p = 0.01), posterior acoustic enhancement (OR = 2.09, p = 0.004), and no calcifications (OR = 2.11, p = 0.005). The combination of these four features showed good specificity (98.4%) and negative predictive value (82.1%) for the TN subgroup.

**Table 3:** Univariate logistic regression analysis for the typical sonographic features of TN invasive breast carcinoma.

Univariate logistic regression analysis		
Predictive factors	OR (95%CI)	P value
Regular shape	1.80 (1.11–2.91)	0.017
No spiculated/angular margin	2.19 (1.30–3.79)	0.004
Posterior acoustic enhancement	2.49 (1.53–4.05)	<0.0005
No calcifications	1.95 (1.17–3.26)	0.009

**Table 4:** Multivariate logistic regression analysis for typical sonographic features of TN invasive breast carcinoma.

Multivariate logistic regression analysis						
Independent factors	OR (95%CI)	P value	Sensitivity	Specificity	PPV	NPV
Regular shape	1.73 (1.05–2.85)	0.033	29.8%	80.9%	27.0%	82.9%
No spiculated/angular margin	2.09 (1.19–3.67)	0.01	82.7%	31.4%	22.2%	88.5%
Posterior acoustic enhancement	2.09 (1.26–3.47)	0.004	31.7%	84.3%	32.4%	83.9%
No calcifications	2.11 (1.25–3.55)	0.005	78.8%	34.4%	22.2%	87.3%
Combination of 4 features			9.6%	98.4%	58.8%	82.1%

*PPV: Positive Predictive Value, NPV: Negative Predictive Value*.

**Figure 1:**
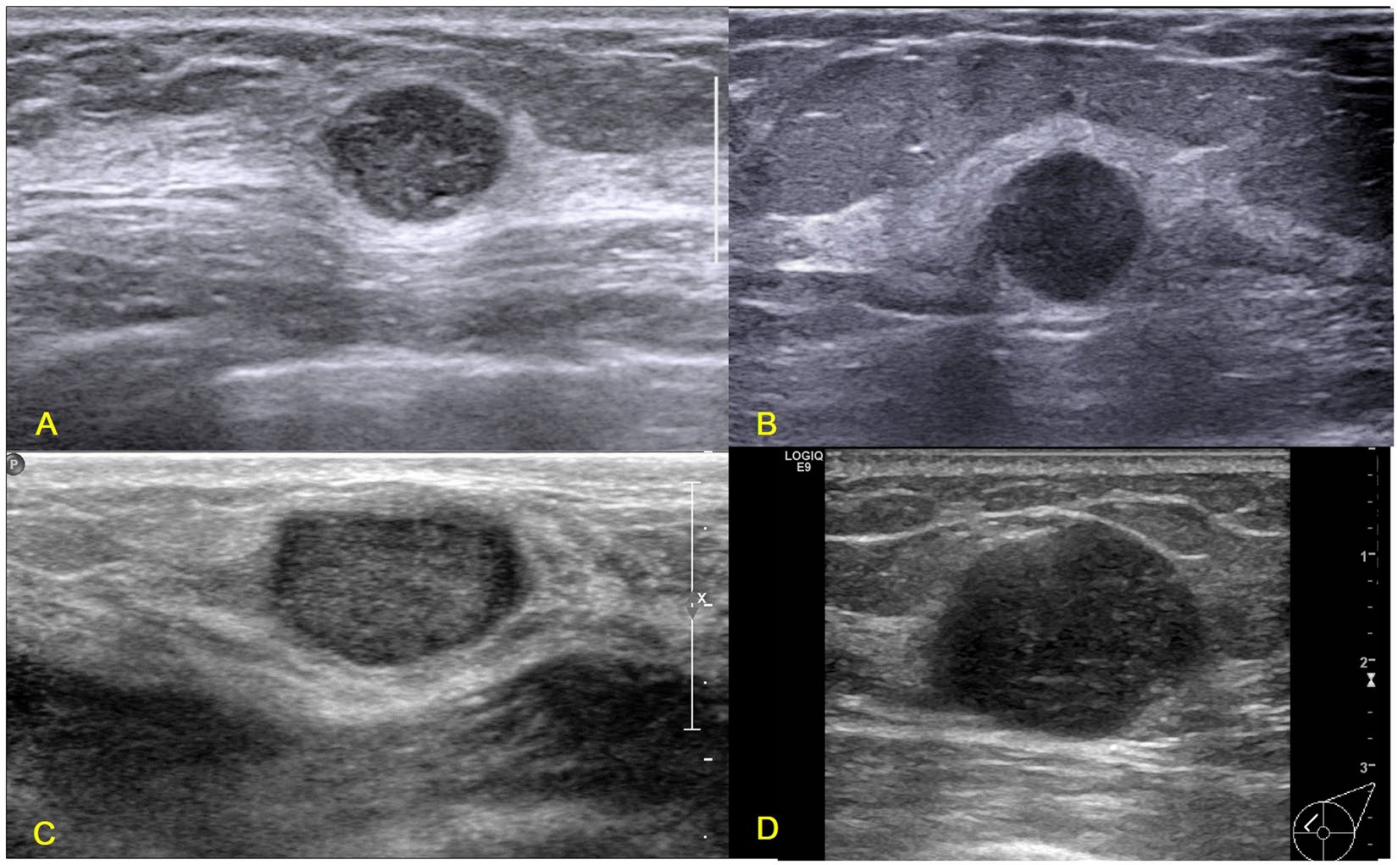
Illustrations of TN breast cancer with regular shape, circumscribed margin and posterior acoustic enhancement. (**A**) Invasive ductal carcinoma in a 35-year-old female patient (BI-RADS: 4A, grade III, Ki67 30%); (**B**) Invasive ductal carcinoma in a 55-year-old female patient (BI-RADS: 4A, grade III, Ki67 30%); (**C**) Invasive ductal carcinoma in a 33-year-old female patient (BI-RADS: 4A, grade III, Ki67 80%); (**D**) Invasive ductal carcinoma in a 48-year-old female patient (BI-RADS: 5, grade III, Ki67 80%).

**Figure 2:**
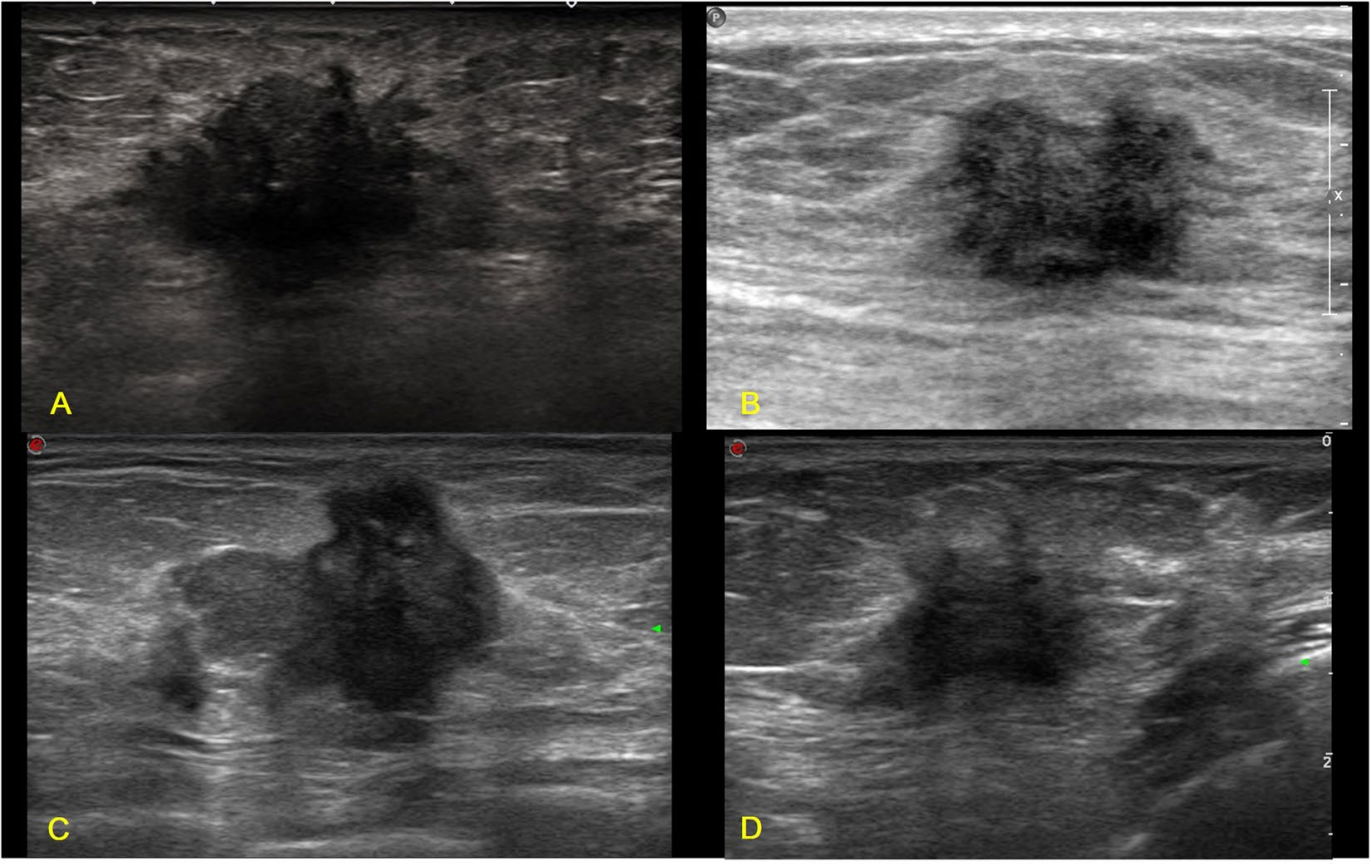
Illustrations of TN breast cancer with irregular shape, spiculated and/or angular margin, and posterior acoustic shadow. (**A**) Invasive ductal carcinoma in a 67-year-old female patient (BI-RADS: 5, grade III, Ki67 25%); (**B**) Invasive ductal carcinoma in a 47-year-old female patient (BI-RADS: 4C, grade II, Ki67 30%); (**C**) Invasive ductal carcinoma in a 69-year-old female patient (BI-RADS: 4C, grade III, Ki67 30%); (**D**) Invasive ductal carcinoma in a 45-year-old female patient (BI-RADS: 4C, grade III, Ki67 80%).

**Figure 3:**
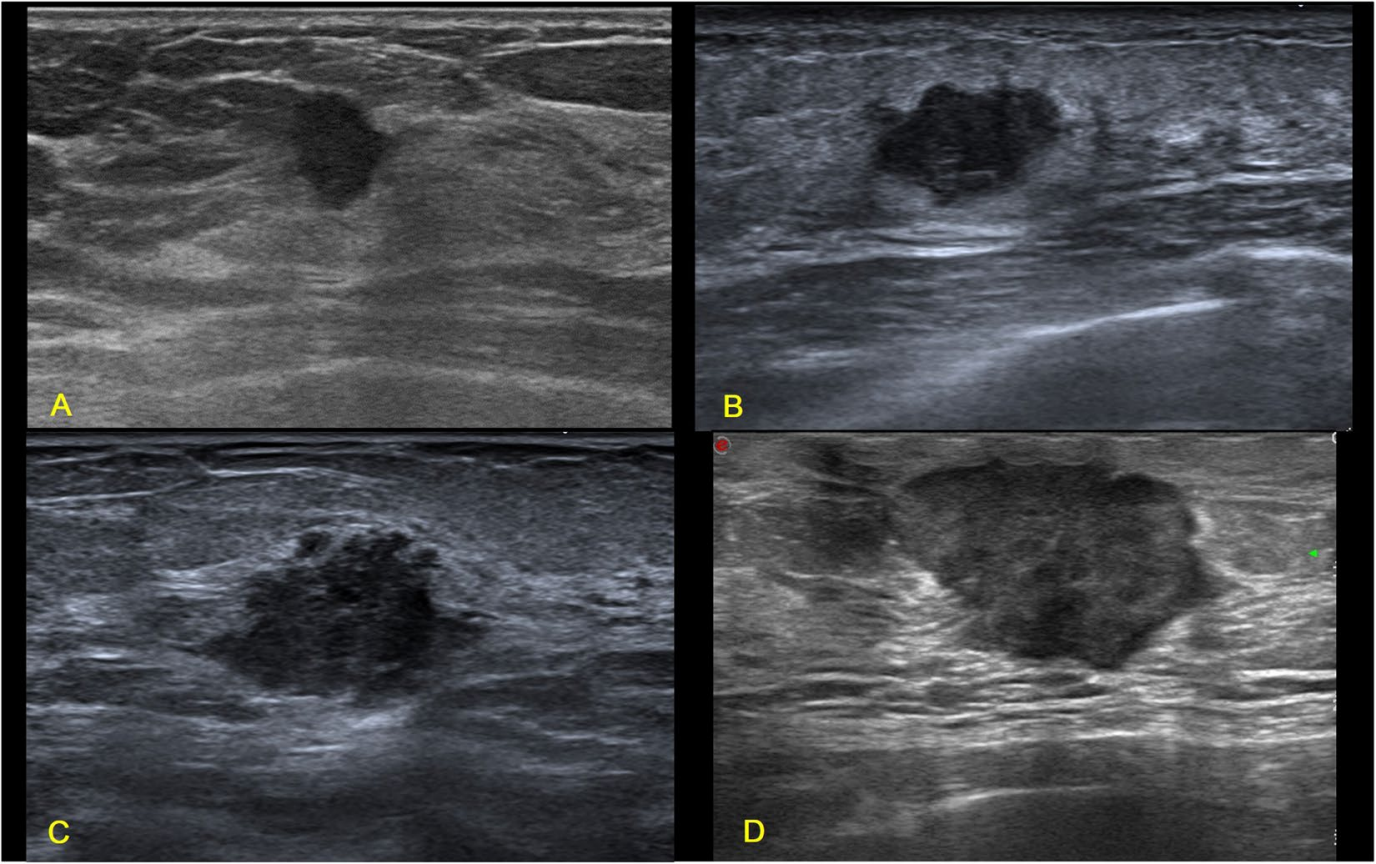
Illustrations of TN breast cancer with irregular shape, spiculated and/or angular margin, and enhancement or no change of posterior acoustic pattern. (**A**) Invasive ductal carcinoma in a 53-year-old female patient (BI-RADS: 4B, grade III, Ki67 30%); (**B**) Invasive ductal carcinoma in a 31-year-old female patient (BI-RADS: 4B, grade III, Ki67 80%); (**C**) Invasive ductal carcinoma in a 70-year-old female patient (BI-RADS: 4C, grade II, Ki67 50%); (**D**) Invasive ductal carcinoma in a 51-year-old female patient (BI-RADS: 4C, grade II, Ki67 20%).

**Figure 4:**
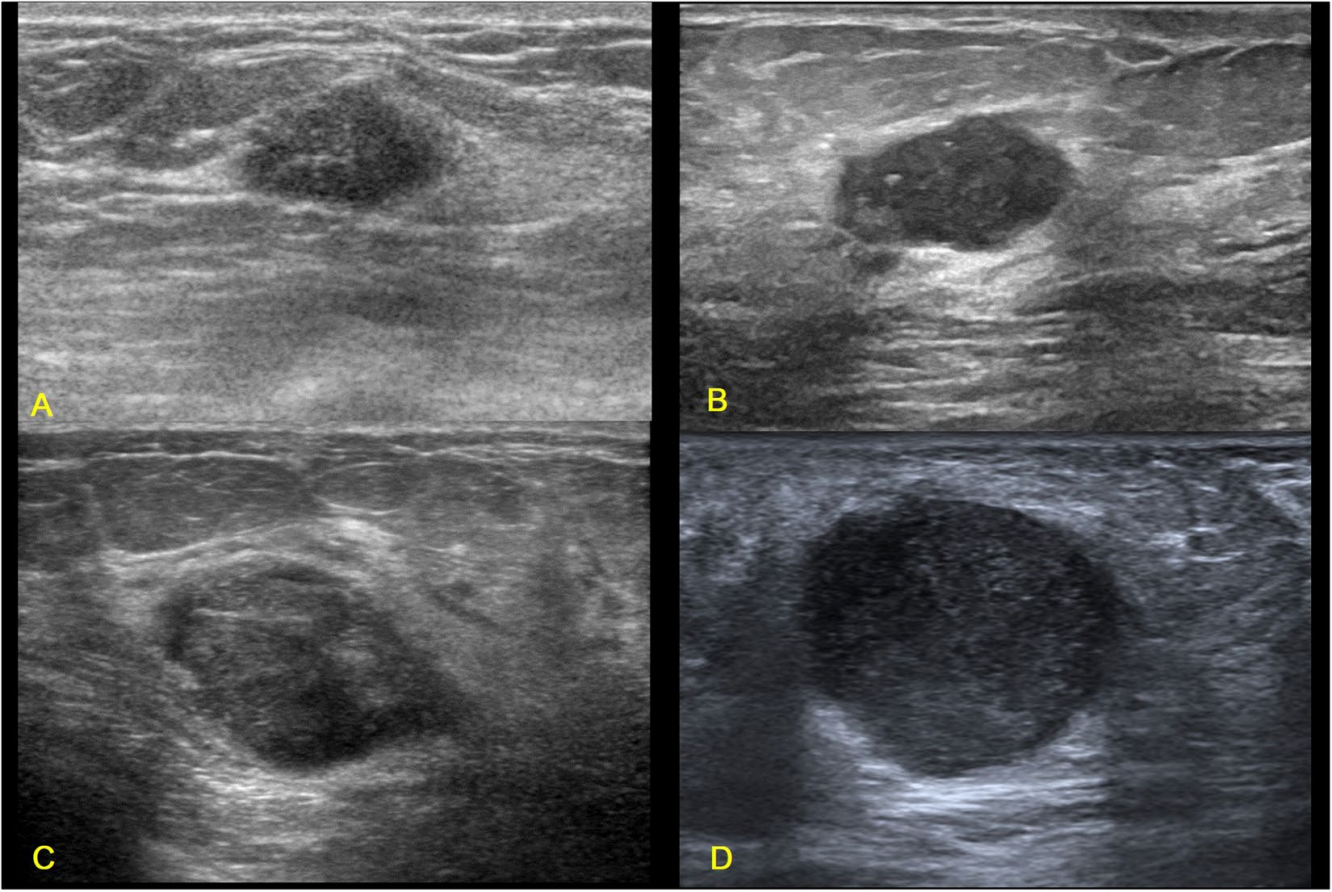
Illustrations of TN breast cancer with uncircumscribed margin but with no spiculated or angular margins. (**A**) Invasive ductal carcinoma in a 49-year-old female patient (BI-RADS: 4C, grade III, Ki67 30%); (**B**) Invasive ductal carcinoma in a 77-year-old female patient (BI-RADS: 4C, grade III, Ki67 70%); (**C**) Invasive ductal carcinoma in a 61-year-old female patient (BI-RADS: 4B, grade III, Ki67 80%); (**D**) Invasive ductal carcinoma in a 66-year-old female patient (BI-RADS: 4C, grade III, Ki67 60%).

**Figure 5:**
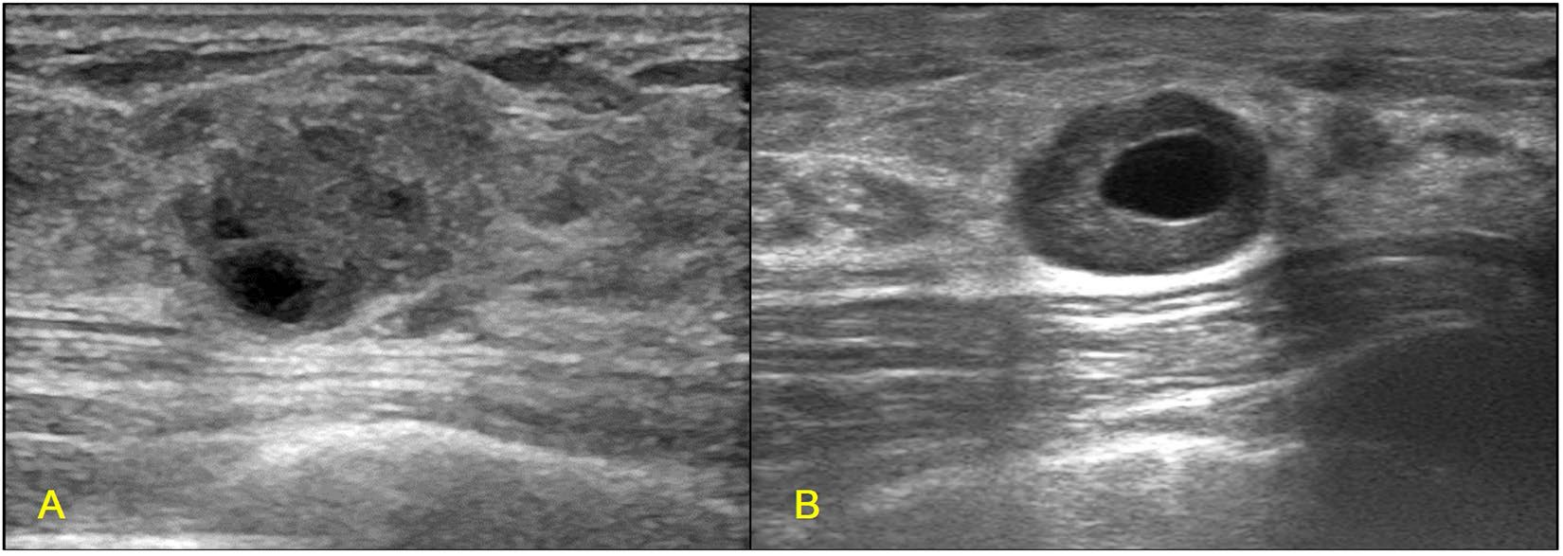
Illustrations of TN breast cancer with complex cystic and solid echo pattern. (**A**) Invasive ductal carcinoma in a 50-year-old female patient (BI-RADS: 4C, grade III, Ki67 80%); (**B**) Invasive ductal carcinoma in a 43-year-old female patient (BI-RADS: 4A, grade III, Ki67 40%).

**Figure 6:**
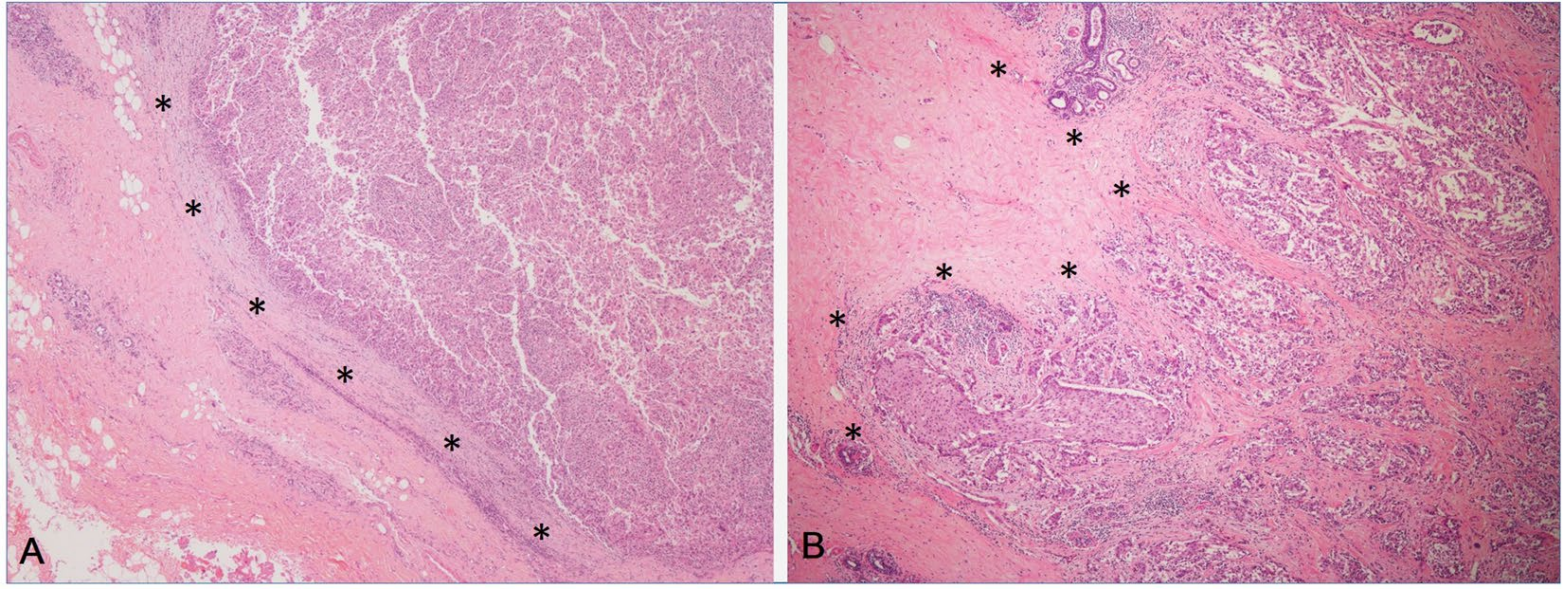
Photomicrograph of the tumor margin (*) of TN breast cancer. (**A**) The circumscribed margin; (**B**) The uncircumscribed margin with cellular prominence (HE stain, original magnification, ×40).

Not all TN breast cancers present the four independent sonographic features. Figure 1 shows a group of TN breast cancers with the presence of all the four independent sonographic features. Figures 2–5 shows the variations of sonographic features of TN breast cancers. Figure 6 demonstrates the circumscribed margin (A) and the uncircumscribed margin (B) upon pathological images. Table 5 shows the associations between clinicopathological factors and sonographic appearances of TN breast cancer. High grade TN breast cancer had more chance to be regular in tumor shape compared with low grade TN breast cancer (p = 0.012). TN breast cancer with high Ki67 expression had more chance to be regular in tumor shape (p = 0.023) and without angular/spiculated margin (p = 0.005). The higher HER2 score was associated with higher chance of calcifications in TN breast cancer (p = 0.033). However, age and tumor size had no effect on the sonographic features of TN breast cancer. Using multivariate logistic regression analysis, high pathological grade tended to be the independent factor for regular shape of TN breast cancer (OR = 7.24, p = 0.064), and high Ki67 expression (≥40%) was the independent factor for no angular/spiculated margin (OR = 4.72, p = 0.014).

**Table 5:** Effect of clinical, pathological and immunohistochemical factors that may affect the sonographic features of TN invasive breast carcinoma. Data are presented as frequency (%).

	Shape		Angular/spiculated margin		Posterior acoustic enhancement		Calcifications	
	Regular	Irregular	Yes	No	Yes	No	Yes	No
**Age**								
<45 yrs	8 (28.6)	20 (71.4)	2 (7.1)	26 (92.9)	12 (42.9)	16 (57.1)	4 (14.3)	24 (85.7)
45–60 yrs	16 (33.3)	32 (66.7)	8 (16.7)	40 (83.3)	16 (33.3)	32 (66.7)	10 (20.8)	38 (79.2)
>60 yrs	7 (25)	21 (75)	8 (28.6)	20 (71.4)	5 (17.9)	23 (82.1)	8 (28.6)	20 (71.4)
**P value**	0.735		0.104		0.126		0.423	
**Tumor size**								
<2 cm	13 (30.2)	30 (69.8)	4 (9.3)	39 (90.7)	14 (32.6)	29 (67.4)	9 (20.9)	34 (79.1)
2–5 cm	18 (30)	42 (70)	14 (23.3)	46 (76.7)	18 (30)	42 (70)	13 (21.7)	47 (78.3)
>5 cm	0 (0)	1 (100)	0 (0)	1 (100)	1 (100)	0 (0)	0 (0)	1 (100)
**P value**	0.807		0.161		0.325		0.87	
**Pathological grade**								
Low	1 (5.6)	17 (94.4)	4 (22.2)	14 (77.8)	6 (33.3)	12 (67.7)	4 (22.2)	14 (77.8)
High	29 (35.4)	53 (64.6)	12 (14.6)	70 (85.4)	26 (31.7)	56 (68.3)	17 (20.7)	65 (79.3)
**P value**	**0.012**		0.426		0.893		0.888	
**Ki 67 level**								
<40%	2 (9.5)	19 (90.5)	8 (38.1)	13 (61.9)	8 (38.1)	13 (61.9)	6 (28.6)	15 (71.4)
≥40%	29 (34.9)	54 (65.1)	10 (12.0)	73 (88.0)	25 (30.1)	58 (69.9)	16 (19.3)	67 (80.7)
**P value**	0.023		**0.005**		0.483		0.352	
**HER2 score**								
0 or 1	29 (32.2)	61 (67.8)	15 (16.7)	75 (83.3)	30 (33.3)	60 (66,7)	16 (17.8)	74 (82.2)
2	2 (14.3)	12 (85.7)	3 (21.4)	11 (78.6)	3 (21.4)	11 (78.6)	6 (42.9)	8 (57.1)
**P value**	0.172		0.661		0.373		**0.033**	

## Discussion

Our study shows that TN invasive breast carcinomas present significant differences in clinical, pathological, and sonographic features compared with non-TN invasive breast carcinomas. Four sonographic features are associated with TN invasive breast carcinoma. However, great diversity of sonographic features was found for the TN invasive breast carcinoma. The pathological grade had significant effect on the tumor shape, the expression level of Ki67 had significant effect on the tumor shape and presence of spiculated/angular margins, and HER2 score had significant effect on the presence of calcifications. Some TN invasive breast carcinomas presented similar sonographic appearances with breast fibroadenomas.

We found that TN invasive breast carcinoma was associated with sonographic features of regular shape, no angular/spiculated margin, posterior acoustic enhancement and no calcifications. This finding was in agreement with previous studies^9,10,12,15,20–24^. These four sonographic features were usually regarded as imaging characteristics of benign breast masses in BI-RADS lexicon. Most malignant breast tumors were used to be believed having an irregular shape with poorly defined spiculated or angular margins and a posterior acoustic shadow^25^. Recently, studies have shown that these malignant-like features are characteristics of low- grade breast cancers with slow cellular proliferation^15,17,18,26^. In contrast, a regular shape with circumscribed margin or posterior acoustic enhancement which were believed to be traits of benign tumors, are often associated with high- grade breast cancers with rapid cellular proliferation^10,17,18^. Our results confirmed these findings that breast tumors with higher pathological grade had more chance to have regular shape; and tumors with higher Ki67 level had less chance of presenting angular/spiculated margin.

It has been described that the smooth appearance of breast tumors are associated with high proliferation rates, which is less likely to induce stromal reactions, and therefore are pushing the borders between the mass and the normal surrounding tissue^13,18,27^. The uncircumscribed margin is believed to be associated with a low proliferative rate of the breast tumor, which gives enough time for stromal interactions. These interactions result in fibrosis, comprising fibroblasts, inflammatory cells, proliferating vascular structures, and normal parenchymal cells surrounding the invasive edge^13,18,27^. The fibrosis also brings out excessive sound reflection or attenuation which results in posterior acoustic shadow in ultrasound images. In contrast to the breast cancers with a posterior acoustic shadow, tumors with posterior acoustic enhancement due to the reduced attenuation of ultrasound energy, were found to be more cellular and tended to be high-grade tumors^28^.

We found that the higher expression level of HER2 in TN breast cancer was associated with the presence of calcifications upon ultrasound images. This phenomenon was addressed in previous studies that HER2 amplification phenotype breast cancer was highly associated with the presence of calcifications compared with other phenotypes^14,29^. However, there was no comparable results for the TN subgroup breast cancer. HER2 gene amplification in breast cancer is associated with increasing tumor cell proliferation, accelerating angiogenesis and reducing apoptosis^30^. The mechanism for the association between HER2 expression level and the presence of calcifications upon ultrasound images in breast cancers is warranted for future study.

While being recognized as an aggressive disease, TN breast cancer is highly diverse among patients with variable clinical outcomes^31–33^. Similarly, the sonographic features of TN breast cancer showed great variations as shown in Figs 1–4. Some TN breast cancers present complex cystic and solid echo as shown in Fig. 5. In addition, as shown in Table 2, the incidence of typical sonographic features for TN breast cancer is not close to 100%, which is consistent with earlier reports^10,13–16,18^. Zhang *et al*., in their study of 1000 cases, found that the incidence of circumscribed margins for the TN group was about 28.7%^14^. These variations of sonographic features of breast cancers have been addressed previously using automated breast ultrasound, and the authors concluded that the prediction value of molecular subtypes using sonographic appearances is limited^34^. Our findings also support this statement due to the variations of sonographic appearances of breast cancers.

Meanwhile, we evaluated the effect of clinical, histological and immunohistochemical factors on the sonographic features of TN breast cancer. Tumor size and age had no effect on the sonographic appearances of TN breast cancers. Ki67 and pathological grade were associated with the tumor shape and tumor margin. However, Ki67, a kind of proliferation marker, is only partially accounted for the variations of TN breast tumor margin according to our data (Table 5). There is a lack of data in the literature to explain these variations of sonographic features for TN breast cancers. Research on breast cancer now is targeting at identifying more prognostic, predictive and therapeutic determinant for the purpose of tailoring personal treatment^35^. The recent advances showed that TN breast cancer can be divided to four subgroups based on cytokeratins^31^, transcriptomes^33^ or genomics^36^ which are associated with the clinical behaviors of TN breast cancers. There is a similar case that the pattern of tumor border upon pathology was associated with the biological subgroups of TN breast cancer^31^. Infiltrative pattern was more associated with Luminal cluster and pushing pattern was more associated with Basal cluster^31^. Another gene expression profile even identified 6 subgroups of TN breast cancers^37^. We postulate the diversity of these biological characteristics to be expected to explain the sonographic variations of TN breast tumors. The association between the sonographic appearance of TN breast cancer and these potential genetic and prognostic determinants is unknown. We will try to address these associations in the future study.

**Figure 7:**
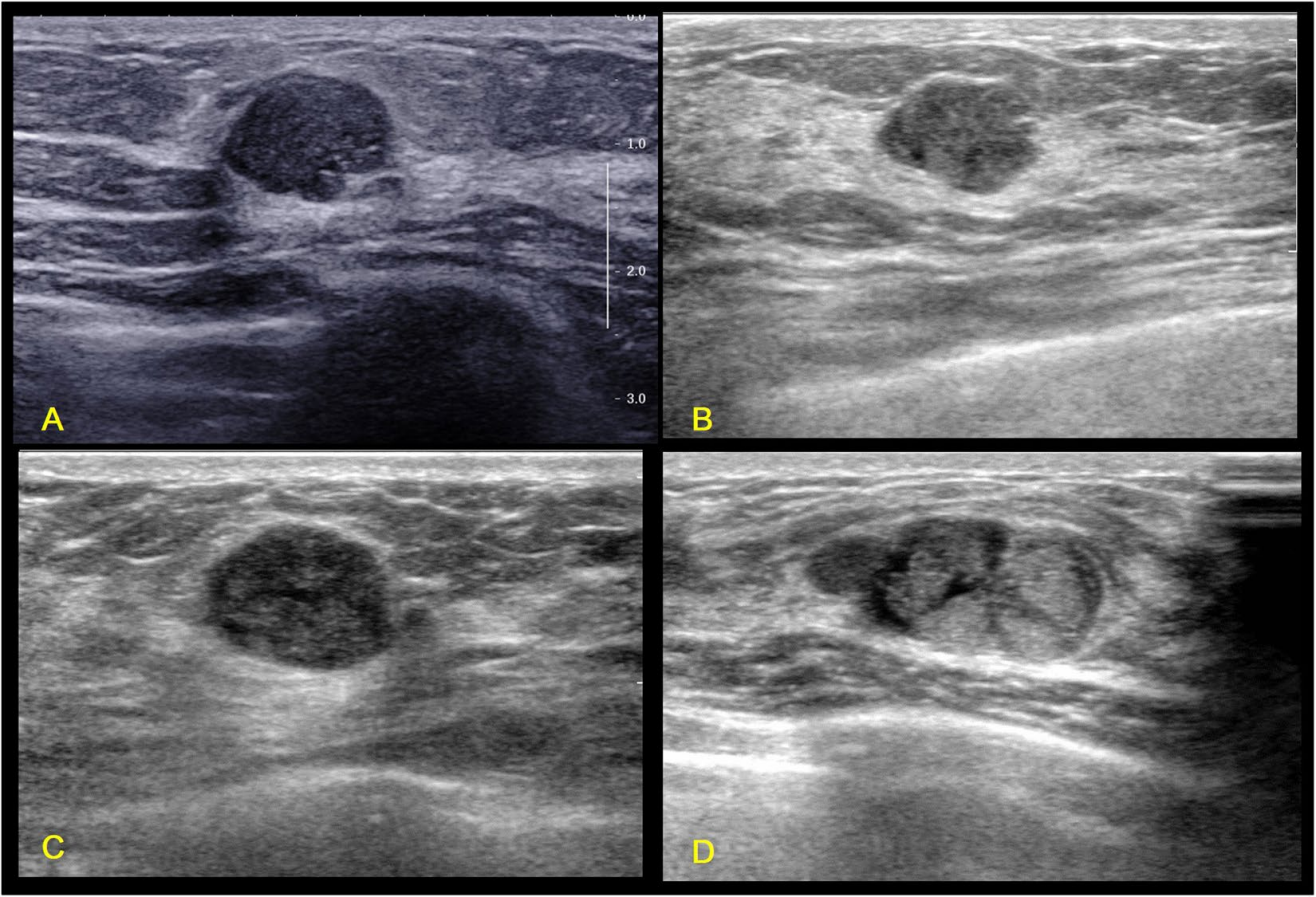
Illustrations of fibroadenomas with TN breast cancer-like sonographic features. (**A**) Fibroademoma in a 37-year-old female patient (BI-RADS: 4B); (**B**) Fibroademoma in a 53-year-old female patient (BI-RADS: 4A); (**C**) Fibroademoma in a 43-year-old female patient (BI-RADS: 4A); (**D**) Fibroademoma in a 27-year-old female patient (BI-RADS: 4A).

**Figure 8:**
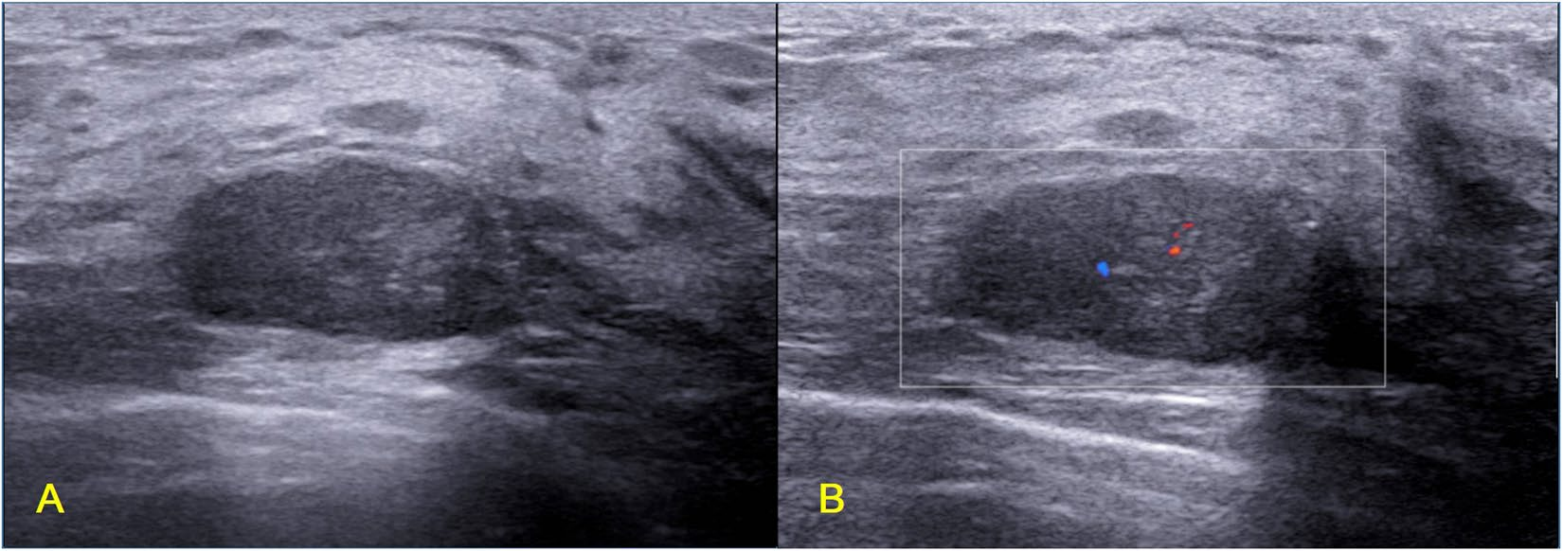
TN invasive ductal carcinoma with fibroadenoma-like sonographic features in a 25-year-old female patient (BI-RADS: 3, grade III, Ki67 90%). (**A**) B mode ultrasound shows a mass with paralleled orientation, regular shape, circumscribed margin and posterior acoustic enhancement; (**B**) Color Doppler ultrasound shows sparse blood flow signal.

Some breast masses with benign sonographic appearances are very difficult to be identified, as shown in Figs 1 and 7. Both groups of breast masses have similar sonographic appearances but with controversial pathological results. Figure 8 shows a TN invasive ductal carcinoma (grade III, Ki67 90%) in a 25-year-old female whose mother suffered from breast cancer. The mass was detected by ultrasound examination occasionally, and it was suspected as fibroadenoma at the outpatient clinic. The mass had paralleled orientation, regular shape, circumscribed margin, posterior acoustic enhancement, and sparse blood flow signal. The BI-RADS score for the mass was grade 3 given by the sonographic physician. However, local resection of the mass proved to be TN invasive breast carcinoma with high pathological grade and high proliferation rate. Finally, breast conservative surgery and sentinel lymph node biopsy was performed for this young patient. The early suspicion and recognition for this kind of benign-look TN breast cancers may benefit the patient in terms of improving prognosis. Therefore, these pathological variations of breast masses call for intense attention for the differential diagnosis of TN breast cancers and benign breast masses especially for young patients. At outpatient clinic, quick and accurate decision for those benign-like breast masses is quite challenging as compared to elderly patients as young patients are usually less likely to be expected to have malignant breast tumors. Therefore, benign-like breast masses with an oval shape and without angular or spiculated margin in young patients require additional attention during ultrasound examinations, particularly by less experienced doctors. The challenge for sonographic physicians calls for more advanced methods to improve diagnostic performance. Radiomics imaging is expected to be an option for improving the diagnostic performance by extracting more invisible characteristics from the ultrasound images^38^. The quantitative ultrasound radiomics imaging has been used to monitor the treatment response of breast cancers^39^. The diagnostic value of ultrasound radiomics used for the differentiation between fibroadenoma and TN breast cancer will be evaluated.

Our results should be interpreted after considering the limitations. First, our study was based on retrospectively reviewed still images. Although we had two observers, it was very likely to miss some information or misinterpret the stored images. In the future, a prospective study with a video loop stored for image assessments is planned. The quantitative assessment using computer-aided technology is also expected to reduce the subjectivity of reading images. Second, our ultrasound data were lack of information on blood flow and elasticity measurement due to the various ultrasound machines used. Third, our results may not be applicable for non-invasive breast carcinomas and some very large breast tumors, which were excluded from our study as they were treated with neoadjuvant chemotherapy before surgery.

TN breast cancers had typical sonographic features. Variations of sonographic features are associated with the pathological grade, Ki67 proliferation level and HER2 score. Some TN breast cancers are easily to be misdiagnosed as benign breast masses especially for young patients. Ultrasound physicians should be aware of these associations and variations for the early and accurate diagnosis of TN breast cancer.
