# Author Correction: Triple-negative invasive breast carcinoma: the association between the sonographic appearances with clinicopathological feature

**DOI:** 10.1038/s41598-020-61260-3

**Published:** 2020-03-06

**Authors:** Jia-wei Li, Kai Zhang, Zhao-ting Shi, Xun Zhang, Juan Xie, Jun-ying Liu, Cai Chang

**Affiliations:** Department of Medical Ultrasound, Fudan University Shanghai Cancer Center, Department of Oncology, Shanghai Medical College, Fudan University, Shanghai, 200032 China

Correction to: *Scientific Reports* 10.1038/s41598-018-27222-6, published online 13 June 2018

The original version of this Article contained errors in the Abstract.

“The independent sonographic features for the TN subgroup included regular shape (odds ratio, OR  =  2.14, p  =  0.007), no spiculated/angular margin (OR  =  1.93, p  =  0.035), posterior acoustic enhancement (OR  =  2.14, p  =  0.004), and no calcifications (OR  =  2.10, p  =  0.008).”

now reads:

“The independent sonographic features for the TN subgroup included regular shape (odds ratio, OR = 1.73, p = 0.033), no spiculated/angular margin (OR = 2.09, p = 0.01), posterior acoustic enhancement (OR = 2.09, p = 0.004), and no calcifications (OR = 2.11, p = 0.005).”

Additionally, this Article contained an error in section (B) of the Figure 6 legend.

“**(B)** The uncircumscribed margin with cellular prominence (HE stain, original magnification, ×4).”

now reads:

“**(B)** The uncircumscribed margin with cellular prominence (HE stain, original magnification, ×40).

